# Virtual Implant Rehabilitation of the Severely Atrophic Maxilla: A Radiographic Study

**DOI:** 10.3390/dj8010014

**Published:** 2020-02-02

**Authors:** Michele Manacorda, Bianca Poletti de Chaurand, Alberto Merlone, Giulia Tetè, Francesca Mottola, Raffaele Vinci

**Affiliations:** Department of Dentistry, IRCCS San Raffaele Hospital and Dental School, Vita Salute University, via Olgettina n.48, 20123 Milan, Italy

**Keywords:** oral surgery, implant planning, maxillary atrophy, implant rehabilitation, CBCT, tilted implants

## Abstract

Background: Advanced maxillary atrophy is one of the most common clinical scenarios where implant placement could become difficult. Nevertheless, a volumetric evaluation using a suitable diagnostic software could facilitate the implant planning. The purpose of the present study is to suggest the potential application of the maxillary retro-canine area as the designated location for virtual tilted implants. Methods: A sample of Cone Beam Computed Tomography (CBCT) images from the Department of Dentistry (IRCSS San Raffaele, Milan, Italy) was evaluated. After a 3D anatomical evaluation, tilted implants were virtually positioned in the retro-canine regions. All the implants were inserted using the same procedure at 30° and 45° degrees of tilting. The length, palatal angulation and diameter of the placed implant were identified. Results: A total of 220 tilted implants were placed. An average implant measurement of 13.51 mm in length and 3.42 mm in diameter were calculated. Additionally, an average buccal–palatal angulation of 6° was identified. Upon statistical analysis, the implant length was found to be significantly higher at 45° degrees of mesio-distal angulation than at 30° degrees (<0.0001). Conclusions: A considerable number of patients show a significant degree of bone atrophy. The implant-supported treatment plan can rely on three-dimensional imaging of the residual bone as a guiding tool to establish the most effective implant position for each specific case. In this study, it was found that an implant could have a greater length if its mesio–distal angulation was more accentuated. In addition, owing to the volumetric evaluation, it was possible to virtually insert tilted implants in almost all of the cases of atrophy. This could lead clinicians to consider the retro-canine area as a viable place to insert a longer tilted implant.

## 1. Introduction

The growing aging population and the psychosocial perception of tooth loss have both contributed to the increase in implant-supported solutions [1,2,3]. Throughout the past twenty years, the use of osseointegration methods has grown more and more critical for oral rehabilitation, to completely regain functions and improve aesthetics [4,5]. This is also due to the frequency of disapproving patient outcomes with the use of removable partial or total dentures [6,7]. One of the most important requirements for dental implants’ osseointegration is the presence of a sufficient amount of basal bone [8]. Unfortunately, totally or partially edentulous maxillae often show a significant degree of sinus pneumatization and/or alveolar bone atrophy [9,10]. This lack of bone may complicate the implant’s placement and could influence the final results [11]. For these reasons, several aspects should be considered before an implant surgery and a 3D volume bone evaluation is essential to plan a proper implant rehabilitation [12,13].

The Cone Beam Computed Tomography (CBCT) produces three-dimensional reconstructions of maxillary and mandibular anatomical structures using a single scan and offering multiple views with low radiations. A flat detector conducts the image capture. The X-ray diffusion is cone-shaped. The CBCT scans allow for a better understanding of the jaws’ morphology and to evaluate the volume of remaining bone in any given site, particularly when considering an implant rehabilitation for immediate load [14,15]. Furthermore, the scans allow interactive planning using 3D simulation software. To date, according to the American Academy of Oral and Maxillofacial Radiology, CBCT should be considered as the method of choice for the three-dimensional evaluation of the maxillary bone to plan an implant treatment [16]. In particular, volumetric data acquired by CBCT showed a high accuracy of the measurements with a relative error below 1% compared with the same measurements taken in vivo as demonstrated by Veyre-Goulet et al. [17].

Many surgical techniques are suitable to place and load implants in edentulous atrophic maxilla. Treatment plans may include bone grafting techniques to reconstruct the lost bone volume [18]. For example, procedures such as distracting osteogenesis, Le Fort osteotomy with inlay grafting, onlay bone grafts, maxillary sinus floor elevation or guided bone regeneration have been used to re-establish bone bulk before implant surgery [19,20,21,22]. However, these methods are characterized by high morbidity, long duration, and high resilience to therapy [23,24,25]. Widmark et al. [26] found that implants inserted in native bone had a higher success rate than implants placed into grafted bone. The zygomatic implants are viable alternatives for treatment of the severely atrophic maxilla, nevertheless, this procedure is operator-dependent [27].

In this study, the insertion of tilted implants is simulated on CBCT scans of atrophic upper jaws in order to calculate the average angulations and measures of the fixtures. The aim of the present paper is to show the potential application of the maxillary retro-canine region as the designated place for tilted “iuxtameatal” implants. “Iuxtameatal” means that the apex of the implants is surrounded by the cortical bone near the nasal inferior meatus. This virtual procedure could facilitate surgeons to find the most effective implant measures and position in order to get more implant stability. In particular, virtual implant insertion could help devise the tilted implant rehabilitation without the need of sinus lifts or invasive bone grafting operations in patients presenting with a severe maxillary atrophy.

## 2. Materials and Methods

### 2.1. Study Population

Patients were selected among those referred for a Cone Beam Computerized Tomography (CBCT) to the Department of Dentistry, IRCCS San Raffaele Hospital, Milan, Italy from December 2015 to January 2018.

Patients were included according to the following criteria:

- Age > 18 years old
- Edentulous mid-maxilla area (at least from the second molar to the canine)
- Atrophic maxilla with a residual flat or depressed ridge form with less than 5 mm in height (i.e., from the lowermost point of the maxillary sinus floor and the most coronal point of the residual crest)

Patients were not included if they presented one of the following exclusion criteria:

- Radiolucent or radiopaque images in the mid-maxilla area
- Implant or impacted tooth in the mid-maxilla area

**Figure 1:**
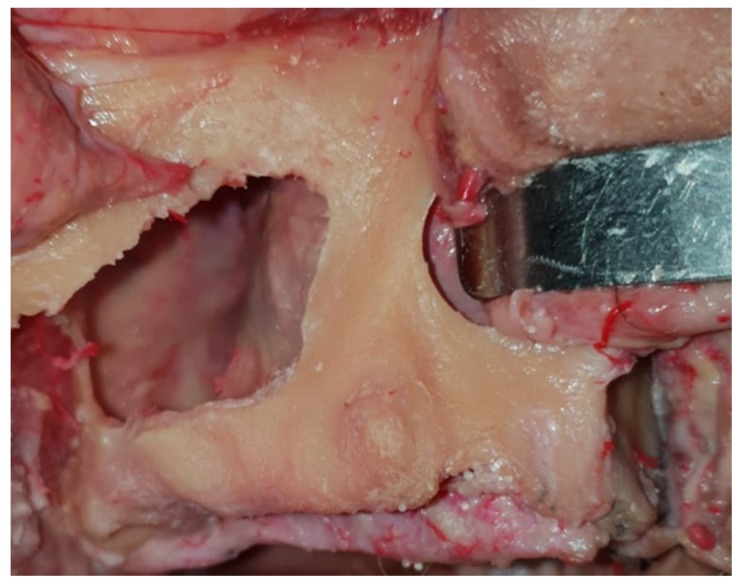
The mid-maxilla region is shown in this fresh frozen ex vivo fixed specimen. The vestibular wall of the right sinus has been removed in order to expose the extension of the paranasal cavity. The residual alveolar bone around the canine region is the area of interest for tilted implant positioning.

The “mid-maxilla” region is an anatomical district that belongs to the maxillary bone (Figure 1).

It is extended from the lateral nasal cavity’s wall until the medial wall of the maxillary sinus cavity, also including the residual alveolar process below the sinus floor. The mid-maxilla could also be identified with the retro-canine bone triangle [28,29].

Each patient provided written consent before undergoing the CBCT scan. All CBCT scans were acquired with a field of view of 12 × 8 cm, at 90 kV 10 mA 16 s and 0.2 mm Voxel size with a NewTom VGi evo Cone Beam 3D Imaging Device (Cefla SC, Imola, Italy). The default position and orientation of the orthogonal sectional planes relative to the jaws were consistent in all the CBCT datasets of each patient. To guarantee a stable head position, all CBCT scans were checked and re-orientated to place the scan view parallel to the Camper’s plane.

### 2.2. Virtual Iuxtameatal Implant Positioning

**Figure 2:**
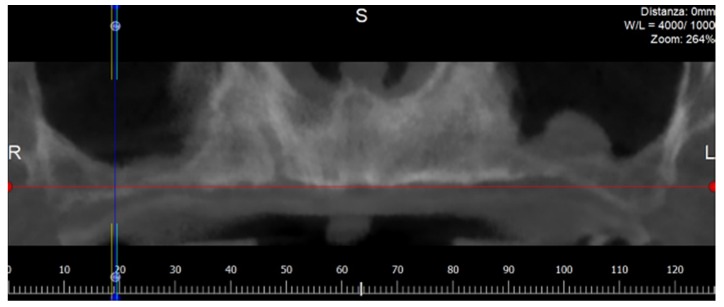
Panorex image elaborated by the software from a maxillary Cone Beam Computed Tomography (CBCT). L: Left; R: Right; S: Supper; L: Lower.

The residual bone of each scan was analyzed using the panoramic reconstruction of the maxilla (Figure 2).

According to the software procedures, the panoramic image was obtained, drawing a panoramic curve on the axial view of the maxillary segment above the residual alveolar crest. The cross-sectional views perpendicular to the panoramic curve were automatically elaborated by the software.

**Figure 3:**
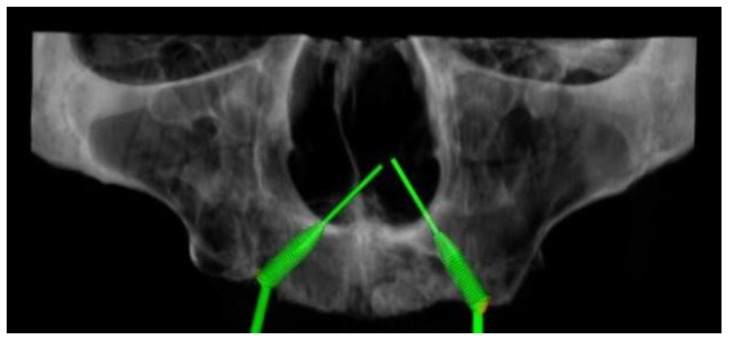
In this 3D reconstruction, the iuxtameatal implant was placed on the right maxilla with an angulation of 45°, and on the left maxilla, with an angulation of 30°.

For each patient, two iuxtameatal cone-shaped implants were virtually placed in a tilted position on each side, at 30° and at 45° degrees of mesio–distal angulation (Figure 3).

**Figure 4:**
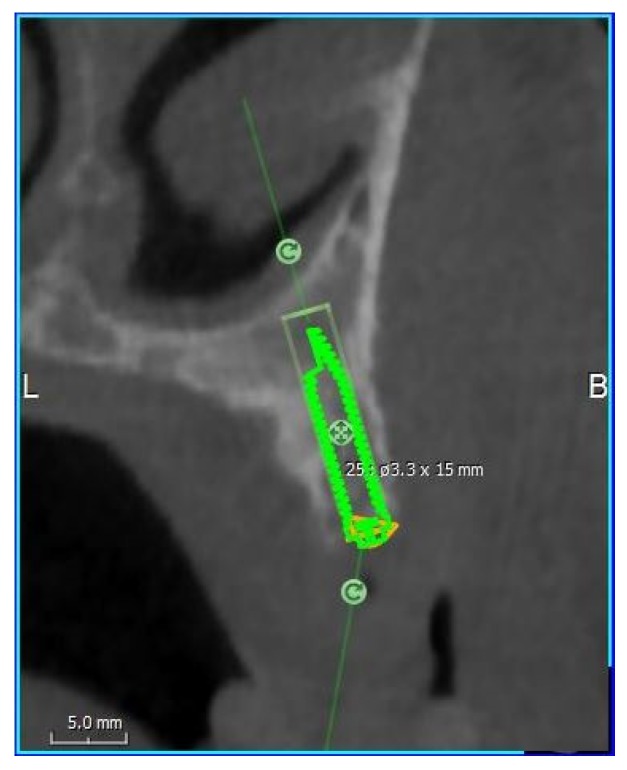
Cross-sectional view during the iuxtameatal implant positioning on the left side with 2 mm of bone surrounding the fixture. B: Buccal; L: Lingual.

Implant insertions were simulated with the RealGUIDE 5.0 implant planning software (3DIEMME, Cantù, Italy). The majority of implant companies produces abutments, which can correct an angle of 30° maximum, but the prosthesis meso-structure can correct an additional 15° of angulation with its conic component, so that the maximum angulation is 45°. All implants were placed in an analogous way, according to the all-on-four procedure described by Malò et al. [30], which attempts to maximally exploit all the bone volume offered, with at least 2 mm of bone surrounding the fixtures (Figure 4).

### 2.3. Outcome Measures

Once the iuxtameatal implants were positioned in the mid-maxilla, the length and the diameter of the implants were measured. The buccal–palatal angulations of each implant were also analyzed in the cross-sectional views. A negative angle indicated that the implant insertion went from the palatal side to the vestibular side of the residual alveolar crest. With the dedicated software, the bone density around the implants’ apex was also measured in gray-scale. All measurements were performed using a digital ruler at 0.1 mm increments by a single trained examiner (A.M).

### 2.4. Statistics

All statistical analyses were performed with a specific software (R, R Core Team, Foundation for Statistical Computing, Vienna, Austria). To evaluate the effect of the tilting degree (30° vs. 45° degrees) on the implant length, a linear mixed-effects model was estimated. In particular, the Linear and Nonlinear Mixed Effects model package was used to estimate the Linear Mixed Effects model (Pinheiro J., Bates D., DebRoy S., and Sarkar D. R Core Team package version 3.1-137 2018).

The modelling approach applied here allows for repeated-measure data and for unobserved heterogeneity among patients to be suitably accounted for. Along with fixed effects, the model allows random components to be specified in the model. An initial complete model was estimated—including position and tilting degree as fixed effects along with their interaction. A subject-specific random effect was specified. Hence, a random intercept model was considered. Assumptions for the correct application of the model were checked. A backward stepwise procedure was applied to select a more parsimonious model. In all the analyses, the significance threshold was set at 0.05.

## 3. Results

CBCT scans of 59 subjects (28 male and 31 females; mean age: 64.5 ± 8.2 years) were included in this study according to the inclusion criteria. Only two scans showed a very severe maxillary atrophy in both the right and left maxillae. Among the other 57 scans, one of them presented the right maxilla as completely reabsorbed and three presented the left maxilla as being too narrow to place any implant. A total of 220 iuxtameatal implant insertions were simulated (see Supplementary Materials Table S1). Average implant measurements of 13.51 mm in length and 3.42 mm in diameter were calculated (Table 1).

**Figure 5:**
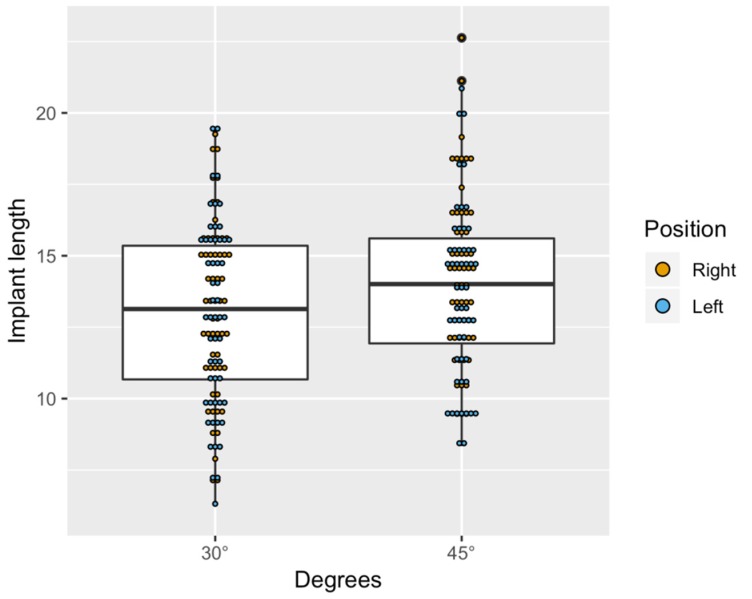
Boxplot showing the distribution of implant length by position and degrees.

In addition, an average anterior–posterior angulation of 6° was identified. In some simulations, the implant axis was negative: the fixtures’ most coronal point was palatal with respect of the apex. The average bone density around implants was 570 gray-scale. After the statistical analysis, the implant length was found to be significantly higher at 45° than at 30° (<0.0001). Thus, when considering the tilting degree of the implant, significant effects of implant length were found (Figure 5).

## 4. Discussion

Krekmanov et al. [31] and Aparicio et al. [32] presented the first papers in which a combination of tilted and axial implants was used in patients with severely reabsorbed posterior maxillae. The results indicate that the use of tilted implants is an effective and safe alternative to maxillary sinus floor augmentation or bone graft procedures. Peñarrocha-Oltra et al. [33], in 2013, wrote that tilted implants, both used alone and combined with axially placed implants and rehabilitated with different prosthetic options have high success rates, minimal complications and high patient satisfaction, even in patients with systemic diseases [34]. Additionally, Balleri et al. [35] presented a very good outcome with 20 fixed partial dentures supported by two implants, one tilted and one axial, in the retro-canine bone triangle. In a recent finite element study for two splinted implants, it appeared that tilting of the distal fixture does not stress the peri-implant bone as compared with the mesial axial fixture [36]. It was also demonstrated that tilted posterior implants were mechanically more advantageous than distal cantilever units [37]. Finally, the systematic review of Wei-Shao Lin [38] demonstrated no differences in implant survival, marginal bone loss, prosthesis survival, or patient-reported outcome measures (PROMs) whether implants are placed axially or with intentional inclination. Nunes et al. analyzed the width and the height of bone volume of the edentulous posterior maxilla using CBCT scans from 122 patients [39]. They found that a high percentage (54%) of molar edentulous sites exhibited a reduced bone height (less than 5 mm) and do require a sinus floor elevation procedure if implant therapy is chosen as a treatment option. However, Nunes et al. did not take into consideration the possibility of tilted implant placement. On the contrary, Tolstunov et al. measured the average bone volume of the edentulous maxilla with cone-beam computerized tomography scans from 30 patients and determined its suitability for implant treatment without additional bone grafting [40]. The results indicated that in many maxillary edentulous cases, the existing bone volume can often be sufficient for a full-arch maxillary implant treatment with tilted implants, without an additional trauma or expense associated with bone grafting or sinus lift. Candel Marti et al. evaluated soft-tissue conditions and bone loss around palatal-positioned implants supporting fixed full-arch prostheses to rehabilitate edentulous atrophied maxillae and compare them with conventional well-centered implants placed in non-atrophic maxillae after a minimum follow-up of 5 years [41]. The results suggested that palatal-positioned implants may be a good treatment alternative for patients with severe horizontal maxillary alveolar bone atrophy.

Owing to mechanical and anatomic difficulties, implant treatment in the atrophic maxilla represents a challenge. In this paper, the examiners were able to find enough bone to adequately distribute the virtual tilted implants in all cases except two. This study presents some limitations, such as the retrospective nature of the present analysis and the virtual placement of the implants. Nevertheless, the statistical analysis demonstrates that an implant can have a greater length if its angulation is more accentuated. Hence, tilting implants would allow the fixture apex to be inserted into the maxillary basal bone, such as the retro-canine region. This area offers the most favorable scope in terms of bone height, width, angulation, and quality when compared with the posterior maxilla [42]. Longer iuxtameatal implants in the mid-maxilla area confer greater implant stability and, eventually, an immediate prosthetic load [43,44]. These tilted implants do not compromise implant placement in the anterior maxilla because they have a marked angulation in the palatal sense. In ortho-panoramic radiography, the correct vestibular–palatal angulation of the implants cannot be planned: only CBCT scans can suggest the ideal angulation with extremely high precision. With the results of this paper, clinicians can be alerted to the importance of the 3D anatomical view to evaluate the amount of cortical bone around the nasal cavity and learn the importance of simulation software to virtually insert implants in severely atrophic maxillae. Among CBCT scans, gray-scales vary widely due to different factors, such as the lack of grey-level uniformity, the presence of artifacts, the effects of scatter and beam hardening [45,46]. On the other hand, different studies demonstrated how grey levels of CBCT can be used to derive Hounsfield units [47]. The gray-scale outcomes reported in this study could suggest that the peri-implant bone density was greater than the average density of the cancellous bone. It indicates that the apex of the iuxtameatal fixtures is effectively inserted into the cortical bone of the walls of the nasal meatus. No studies were found that measured the atrophic maxilla bone volume related to tilted implant treatment. The absence of similar studies in the scientific literature limits the ability to make any comparisons with other studies.

### Hypothetical Complications

The iuxtameatal implants could be affected by mucositis and peri-implantitis, as with any other implants [48]. In order to prevent peri-implant disease, it is recommended that patients undergo an annual professional intervention protocol comprising of mechanical debridement and oral hygiene instructions [49]. Benefitting from the virtual plan previously developed, the implants will unlikely exceed the cortical lamina of the nasal lateral wall. In the eventuality that this happens, the associated risks may be epistaxis, implant displacement in the nasal cavity, rhinitis, or post-operative maxillary cyst [50]. Nonetheless, the validity of this statement needs to be confirmed through future clinical studies.

## 5. Conclusions

A good anatomical background and an accurate tridimensional virtual plan before conducting implant surgery could help clinicians determine the optimal implant angulation and position. Surgeons should take advantage of the mid-maxilla area in particular—the maxillary region with the highest bone density—in order to increase the implant stability. Within the limits of the present study, it can be concluded that inserting an iuxtameatal implant into the mid-maxilla area can be applied to patients with severely atrophic maxillae, except for in some rare cases. Further studies are needed to investigate the clinical use of these iuxtameatal tilted implants.

## Figures and Tables

**Table 1:** Descriptive statistical data for tilted implants at 30° and 45° of angulation.

**30° Tilted Implants**	**N**	**Mean**	**SD**	**Median**	**Min**	**Max**	**Range**
Alveolar crest height	110	4.60	0.39	4.7	2.5	5	2.5
Implant length	110	13.04	3,13	13.17	6.32	19.49	13.17
Implant diameter	110	3.44	0.33	3.35	2.76	4.39	1.63
Palatal angle	110	6.30	7.28	6.80	−24.27	31.52	55.79
Bone quality	110	570	126.17	563.5	274	934	660
**45° Tilted Implants**	**N**	**Mean**	**SD**	**Median**	**Min**	**Max**	**Range**
Alveolar crest height	110	4.60	0.39	4.7	2.5	5	2.5
Implant length	110	13.98	2.96	14.07	8.34	22.63	14.29
Implant diameter	110	3.39	0.32	3.36	2.62	4.07	1.45
Palatal angle	110	5.25	7.13	5.87	−21.19	26.72	47.91
Bone quality	110	568.86	128.22	563.5	274	952	678

## Supplementary Materials

The following are available online at https://www.mdpi.com/2304-6767/8/1/14/s1, Table S1: Study Outcomes.

## Abbreviations

CBCT	Cone Beam Computed Tomography
